# Physical injuries as triggers for self-harm: a within-individual study of nearly 250 000 injured people with a major psychiatric disorder

**DOI:** 10.1136/bmjment-2023-300758

**Published:** 2023-06-28

**Authors:** Amir Sariaslan, Henrik Larsson, Keith Hawton, Joonas Pitkänen, Paul Lichtenstein, Pekka Martikainen, Seena Fazel

**Affiliations:** 1 Department of Psychiatry, University of Oxford, Oxford, UK; 2 School of Medical Sciences, Örebro University, Örebro, Sweden; 3 Department of Medical Epidemiology and Biostatistics, Karolinska Institutet, Stockholm, Sweden; 4 Population Research Unit, Faculty of Social Sciences, University of Helsinki, Helsinki, Finland; 5 International Max Planck Research School for Population Health and Data Science, Rostock, Germany; 6 Centre for Health Equity Studies (CHESS), Stockholm University and Karolinska Institutet, Stockholm, Sweden; 7 Max Planck Institute for Demographic Research, Rostock, Germany

**Keywords:** Suicide & self-harm, Schizophrenia & psychotic disorders, Depression & mood disorders

## Abstract

**Background:**

Although there is robust evidence for several factors which may precipitate self-harm, the contributions of different physical injuries are largely unknown.

**Objective:**

To examine whether specific physical injuries are associated with risks of self-harm in people with psychiatric disorders.

**Methods:**

By using population and secondary care registers, we identified all people born in Finland (1955–2000) and Sweden (1948–1993) with schizophrenia-spectrum disorder (n=136 182), bipolar disorder (n=68 437) or depression (n=461 071). Falls, transport-related injury, traumatic brain injury and injury from interpersonal assault were identified within these subsamples. We used conditional logistic regression models adjusted for age and calendar month to compare self-harm risk in the week after each injury to earlier weekly control periods, which allowed us to account for unmeasured confounders, including genetics and early environments.

**Findings:**

A total of 249 210 individuals had been diagnosed with a psychiatric disorder and a physical injury during the follow-up. The absolute risk of self-harm after a physical injury ranged between transport-related injury and injury from interpersonal assault (averaging 17.4–37.0 events per 10 000 person-weeks). Risk of self-harm increased by a factor of two to three (adjusted OR: 2.0–2.9) in the week following a physical injury, as compared with earlier, unexposed periods for the same individuals.

**Conclusions:**

Physical injuries are important proximal risk factors for self-harm in people with psychiatric disorders.

**Clinical implications:**

Mechanisms underlying the associations could provide treatment targets. When treating patients with psychiatric illnesses, emergency and trauma medical services should actively work in liaison with psychiatric services to implement self-harm prevention strategies.

**WHAT IS ALREADY KNOWN ON THIS TOPIC**

- We identified one relevant study conducted in rural China using monthly data that reported a strong but imprecise estimate for the association between being physically assaulted and attempting suicide (OR: 16.0, 95% CI 2.1 to 120.7).

**WHAT THIS STUDY ADDS**

- To our knowledge, this is the first within-individual study that has examined physical injuries (fall-related, transport-related, traumatic brain injury and injury from interpersonal assault) as potential triggers for self-harm. By pooling nationwide registry data from Finland and Sweden, we were able to study over 1.9 million injured individuals, of whom nearly 250 000 had a lifetime diagnosis of a psychiatric disorder. By comparing each individual with themselves across time, we were able to account for all unmeasured confounders on the individual level that did not vary across the follow-up period (eg, genetics, early environments and neurodevelopmental disorders). We found that people diagnosed with psychiatric disorders were two to three times as likely to engage in self-harm in the week following a physical injury compared with earlier periods, with minimal differences between diagnostic categories. The relative risks were larger for the general population without psychiatric diagnoses, but the absolute risks were low.

**HOW THIS STUDY MIGHT AFFECT RESEARCH, PRACTICE OR POLICY**

- Physical injuries can be precipitants for self-harm in the short term, and hence are potential targets for self-harm prevention, assessment and intervention.

## Introduction

Self-harm is a leading global public health concern^1^ and ranks in the top 15 leading causes of disability-affected life years for individuals between the ages of 10 years and 49 years.^2^ It is a leading risk factor for completed suicide,^3^ which accounts for an estimated 800 000 deaths annually.^4^ Although the clinical presentations of patients who self-harm and those who die by suicide tend to be heterogeneous and have partially distinct aetiologies,^5^ they are typically conceptualised as being on a continuum of suicidal behaviours with varying degrees of severity.^6^ The identification of aetiologically relevant and modifiable risk factors for self-harm is key to developing effective preventive interventions, but evidence has been limited thus far.^7^ Risk factors identified, however, include both predisposing and precipitating factors.^7^ Predisposing factors include, for example, family history of suicidal behaviour^5 8^ and neuropsychiatric disorder.^9^

However, less is known about more proximal factors and in particular recent triggers.^10^ Among these, life events remain influential in models of suicidal behaviours, but empirical work has relied on measuring the aggregated burden of multiple life events rather than targeting specific life events and their timing.^11^ One such life event is physical injury, including, for example, unintentional injuries, traumatic brain injury and injury from interpersonal assault. With regard to these acting as potential triggers for self-harm, a relevant case-crossover study including 1200 participants conducted in rural China found a 16-fold increased odds of suicide attempts during the months when participants reported having been physically assaulted compared with the preceding control month.^12^ However, we are not aware of other relevant studies, especially those of a longitudinal nature. Therefore, detailed information on physical injuries has largely not been included in systematic reviews as risk factors for suicidal behaviour.^7^ Clarifying the evidence for these as potential triggers, as well as the possible moderation of their effects by psychiatric disorders, is important in order to better understand the suicidal process and, especially, whether it may offer novel targets for prevention and intervention.^7^ In addition, results of such investigations may provide clinically useful information to help stratify risk by specific triggers in assessment, which can assist with safety planning and decisions regarding frequency of healthcare contact.

To clarify the role of physical injuries in precipitating self-harm, we conducted a population-based study of physical injuries as triggers using nationwide registry data from Finland and Sweden, which included all individuals exposed to four specific types of physical injuries that resulted in medical care, namely, falls, transport-related injury, traumatic brain injury and injury from interpersonal assault. By adopting a within-individual design, we were further able to quantify risks of self-harm in the week following exposure to any of the physical injuries as compared with earlier weekly control periods within the same person, thus accounting for all time-stable individual-level unmeasured confounders (eg, genetic risks, early-life environmental factors and neurodevelopmental disorders). Additionally, this approach allowed us to assess the moderating role of psychiatric disorders on these triggers.

## Methods

### Data

All Finnish and Swedish residents are assigned a personal identification number, which is used in nationwide registers and provides accurate linkage.

The Care Register for Healthcare, maintained by the Finnish Institute for Health and Welfare, and the National Patient Register, maintained by the Swedish National Board for Health and Welfare, provided data on inpatient hospitalisation episodes (Finland: 1969–2020, Sweden: 1973–2013) as well as specialist outpatient care visits (Finland: 1998–2020, Sweden: 2001–2013). The diagnostic classifications were based on the Finnish and Swedish versions of the International Classification of Diseases (ICD) nomenclature, from the 8th to the 10th revision. Data on mortality dates, including the primary and contributory causes of death, were derived from the causes of death registers in each country, which were based on the same ICD classifications as the patient registries. Emigration dates were derived from the population registers. Data on educational attainment were gathered from the annual administrative registers in both countries (Finland: 1987–2019, Sweden: 1985–2013).

Our sample comprised all persons born in Finland between 1955 and 2000 (n=3 026 607) and in Sweden between 1948 and 1993 (n=4 873 373) who were either registered residents at the end of 1987 in Finland or 1973 in Sweden, or subsequent years if they were born after these dates. We excluded those who had either emigrated (n=101 213) or died (n=8261) before reaching the age of 12 years (ie, the start of the follow-up). Our analytical sample therefore consisted of 7 790 506 individuals.

### Psychiatric disorders and physical injuries

Within the analytical sample, we identified all individuals who had any lifetime diagnoses of either schizophrenia-spectrum disorder, bipolar disorder or depression by using a hierarchical approach (online supplemental etable 1). Throughout the follow-up period (Finland: 1 June 1987–1 December 2020; Sweden: 1 June 1973–1 December 2013), we used hospital records to identify all instances of the following physical injury events (online supplemental etable 1): fall-related injuries, transport-related injuries, traumatic brain injury and injury from interpersonal assault. It should be noted that our definition of injury from interpersonal assault is medical rather than legal, meaning that we cannot determine whether the individuals were legally responsible for the altercations that resulted in their needing medical attention. We excluded physical injury events that occurred within 14 days of each other to avoid duplication of the same episode. The validity of these diagnoses is discussed in online supplemental etext 1.

10.1136/bmjment-2023-300758.supp1 Supplementary data

### Self-harm

We defined self-harm as either a hospital outpatient visit or inpatient episode associated with any diagnosis of intentional self-harm or event of undetermined intent (online supplemental etable 1). Self-harm events of underdetermined intent are commonly included in epidemiological studies of self-harm to avoid misclassification of an estimated quarter of true self-harm events.^5 13^

### Research design

We adopted a time-stratified case-crossover design, similar to an earlier study of ours examining triggers for violent perpetration.^14^ By using this approach, we were able to examine the transient effects of physical injuries as proximal risk markers for self-harm while accounting for an aggregate of all time-stable individual-specific confounders. This was made possible because the rates of self-harm were compared within each individual across time, thus implying that all factors that were constant within the individuals (eg, genetic and early childhood environmental factors, as well as childhood onset neurodevelopmental disorders) were controlled for via the research design. In the present study, we examined the risk of an individual engaging in a self-harm in the week following exposure to any of the physical injuries, and we compared this risk with earlier control periods when they were unexposed to the injury risk marker in question. The control periods had an equivalent follow-up period of a week and were measured every calendar month before the exposure to the physical injury, up to a maximum of 36 months. If the individuals had been exposed to multiple physical injury events, we included them and their respective control periods unless the latter occurred prior to the preceding physical injury event.

### Statistical analysis

We initially fitted separate conditional logistic regression models to the Finnish and Swedish samples to estimate country-specific within-individual associations between physical injuries and self-harm, which were expressed as adjusted ORs (aORs). The models were stratified across individuals and adjusted for age (continuous) and calendar month (categorical, measured at the start of the period) as time-varying confounders. We investigated potential moderation effects by stratifying the models across sex and age (categorical measure; 19 years or younger, 20–29 years, 30–39 years, 40–49 years and 50 years or older). There were no missing data for the measures.

We then estimated pooled estimates of these associations by fitting inverse variance-weighted fixed-effects meta-analysis models to summary statistics data derived from both countries.^15^ In brief, the approach allowed us to pool the within-individual associations by weighting the relative contributions of the country-specific estimates by their sample sizes. We conducted several complementary sensitivity analyses to test the robustness of our findings (online supplemental etext 2).

## Results

Across both countries, we identified 136 182 individuals diagnosed with schizophrenia-spectrum disorders, 68 437 with bipolar disorder and 461 071 with depression. Compared with individuals in the general population without any psychiatric disorders (n=6 566 541), women were over-represented among people with mood disorders (eg, bipolar disorder and depression, 59.6%–60.5% vs 47.8%) but under-represented among people with schizophrenia-spectrum disorders (43.6% vs 47.8%, table 1). People with psychiatric disorders were more likely than the general population without psychiatric disorders to have low educational attainment (18.6%–29.4% vs 9.8%). The rates of having experienced any of the physical injury triggers were higher among those with psychiatric disorders than the general population (36.0%–41.8% vs 25.9%), with the largest absolute differences being for fall-related injuries (24.7%–28.7% vs 18.1%, table 2).

**Table 1:** Sociodemographic factors

	Schizophrenia-spectrum disorder	Bipolar disorder	Depression	No psychiatric disorders
Total				
Finland	78 123	33 404	241 013	2 385 399
Sweden	58 059	35 033	220 058	4 181 142
Pooled	136 182	68 437	461 071	6 566 541
Sex				
Finland				
Women	33 828 (43.3%)	19 119 (57.2%)	146 420 (60.8%)	1 146 451 (48.1%)
Men	44 295 (56.7%)	14 285 (42.8%)	94 593 (39.2%)	1 238 948 (51.9%)
Sweden				
Women	25 492 (43.9%)	21 649 (61.8%)	132 507 (60.2%)	1 992 072 (47.6%)
Men	32 567 (56.1%)	13 384 (38.2%)	87 551 (39.8%)	2 189 070 (52.4%)
Pooled				
Women	59 320 (43.6%)	40 768 (59.6%)	278 927 (60.5%)	3 138 523 (47.8%)
Men	76 862 (56.4%)	27 669 (40.4%)	182 144 (39.5%)	3 428 018 (52.2%)
Immigrant background				
Finland	1568 (2.0%)	644 (1.9%)	5881 (2.4%)	46 814 (2.0%)
Sweden	1579 (2.7%)	1355 (3.9%)	8278 (3.8%)	117 393 (2.8%)
Pooled	3147 (2.3%)	1999 (2.9%)	14 159 (3.1%)	164 207 (2.5%)
Birth year				
Finland				
1948–1950	–	–	–	–
1951–1960	15 128 (19.4%)	5621 (16.8%)	34 734 (14.4%)	375 410 (15.7%)
1961–1970	20 731 (26.5%)	8427 (25.2%)	50 629 (21.0%)	585 507 (24.5%)
1971–1980	15 982 (20.5%)	7132 (21.4%)	43 796 (18.2%)	500 129 (21.0%)
1981–1990	15 918 (20.4%)	7324 (21.9%)	56 462 (23.4%)	485 265 (20.3%)
1991–2000	10 364 (13.3%)	4900 (14.7%)	55 392 (23.0%)	439 088 (18.4%)
Sweden				
1948–1950	6291 (10.8%)	2284 (6.5%)	15 160 (6.9%)	302 760 (7.2%)
1951–1960	19 311 (33.3%)	7316 (20.9%)	46 333 (21.1%)	902 581 (21.6%)
1961–1970	15 338 (26.4%)	8194 (23.4%)	46 054 (20.9%)	970 669 (23.2%)
1971–1980	9017 (15.5%)	7522 (21.5%)	44 988 (20.4%)	863 808 (20.7%)
1981–1990	6962 (12.0%)	7932 (22.6%)	52 914 (24.0%)	844 890 (20.2%)
1991–2000	1140 (2.0%)	1785 (5.1%)	14 609 (6.6%)	296 434 (7.1%)
Pooled				
1948–1950	6291 (4.6%)	2284 (3.3%)	15 160 (3.2%)	302 760 (4.6%)
1951–1960	34 439 (25.3%)	12 937 (18.9%)	81 067 (17.6%)	1 277 991 (19.5%)
1961–1970	36 069 (26.5%)	16 621 (24.3%)	96 683 (21.0%)	1 556 176 (23.7%)
1971–1980	24 999 (18.4%)	14 654 (21.4%)	88 784 (19.3%)	1 363 937 (20.8%)
1981–1990	22 880 (16.8%)	15 256 (22.3%)	109 376 (23.7%)	1 330 155 (20.3%)
1991–2000	11 504 (8.4%)	6685 (9.8%)	70 001 (15.2%)	735 522 (11.2%)
Low educational attainment				
Finland	24 203 (31.0%)	6971 (20.9%)	45 079 (18.7%)	217 457 (9.1%)
Sweden	15 827 (28.2%)	6153 (17.7%)	40 589 (18.6%)	423 397 (10.2%)
Pooled	40 030 (29.4%)	13 124 (19.2%)	85 668 (18.6%)	640 854 (9.8%)

**Table 2:** Rates of triggers occurring during the follow-up period among people with and without psychiatric disorders

	Schizophrenia-spectrum disorder	Bipolar disorder	Depression	No psychiatric disorders
Any trigger				
Finland	27 105 (34.7%)	13 769 (41.2%)	82 308 (34.2%)	544 570 (22.8%)
Sweden	21 875 (37.7%)	14 809 (42.3%)	89 344 (40.6%)	1 155 930 (27.6%)
Pooled	48 980 (36.0%)	28 578 (41.8%)	171 652 (37.2%)	1 700 500 (25.9%)
Fall-related injury				
Finland	18 288 (23.4%)	9514 (28.5%)	56 436 (23.4%)	378 826 (15.9%)
Sweden	15 300 (26.4%)	10 154 (29.0%)	62 055 (28.2%)	806 706 (19.3%)
Pooled	33 588 (24.7%)	19 668 (28.7%)	118 491 (25.7%)	1 185 532 (18.1%)
Transport-related injury				
Finland	6902 (8.8%)	3632 (10.9%)	21 274 (8.8%)	125 837 (5.3%)
Sweden	6771 (11.7%)	4973 (14.2%)	30 160 (13.7%)	352 549 (8.4%)
Pooled	13 673 (10.0%)	8605 (12.6%)	51 434 (11.2%)	478 386 (7.3%)
Traumatic brain injury				
Finland	12 040 (15.4%)	6026 (18.0%)	32 734 (13.6%)	162 090 (6.8%)
Sweden	9163 (15.8%)	5650 (16.1%)	32 462 (14.8%)	332 632 (8.0%)
Pooled	21 203 (15.6%)	11 676 (17.1%)	65 196 (14.1%)	494 722 (7.5%)
Injury from interpersonal assault				
Finland	5271 (6.7%)	2313 (6.9%)	11 666 (4.8%)	32 213 (1.4%)
Sweden	4175 (7.2%)	2575 (7.4%)	14 043 (6.4%)	75 197 (1.8%)
Pooled	9446 (6.9%)	4888 (7.1%)	25 709 (5.6%)	107 410 (1.6%)

**Figure 1:**
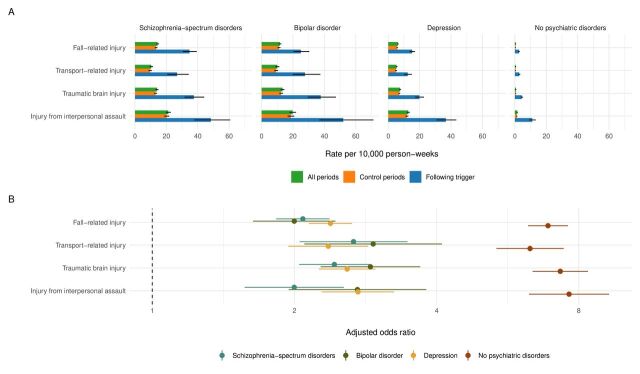
Absolute (A) and relative (B) risks of self-harm across individuals experiencing specific physical injuries (eg, fall-related injury, transport-related injury, traumatic brain injury and injury from interpersonal assault) and psychiatric diagnostic categories (eg, those with schizophrenia-spectrum disorder, bipolar disorder and depression, and those without psychiatric disorders) in Finland and Sweden (n=1 949 710). Notes: The within-individual comparisons were adjusted for age, calendar month and all time-stable individual-level unmeasured confounders (eg, genetic risks, early childhood environmental factors and early-onset neurodevelopmental disorders). The psychiatric disorders were defined hierarchically.

The baseline risk of self-harm (ie, the range of the rates across the control periods) was elevated in people with psychiatric disorders compared with the general population (5.0–20.2 vs 0.4–1.2 per 10 000 person-weeks; figure 1A and online supplemental etable 2). Among individuals with schizophrenia-spectrum disorders, the absolute risk of self-harm was considerably higher following exposure to any of the physical injuries compared with earlier unexposed periods, ranging from transport-related injuries (26.8 vs 9.7 events per 10 000 person-weeks) to injuries from interpersonal assault (48.3 vs 20.2 per 10 000 person-weeks; figure 1A and online supplemental etable 2). Across all categories of individuals with psychiatric disorders and the general population without psychiatric disorders, the absolute rates of self-harm were elevated following any of the trigger events compared with earlier control periods, although the magnitude of the rates varied by psychiatric disorders and injury type (figure 1A and online supplemental etable 2).

Following adjustments for age, calendar month and time-stable unmeasured confounders using within-individual models, these physical triggers were associated with twofold to threefold increased odds of self-harm among people with psychiatric disorders, ranging from injuries from interpersonal assault in people with schizophrenia-spectrum disorders (aOR: 2.0, 95% CI 1.6 to 2.5) to transport-related injuries in those with bipolar disorder (aOR: 2.9, 2.1–4.1; figure 1B). These relative risks were stronger in the general population, with six to eight times increased odds of self-harm compared with earlier unexposed periods within the same individuals (aORs: 6.3–7.6, figure 1B).

Stratifying these within-individual analyses across men and women, we did not find consistent differences across all psychiatric disorders. Injury from interpersonal assault exposure in men with schizophrenia-spectrum disorders was more strongly associated with self-harm than in women with the same disorders (aOR_men_: 2.9, 95% CI 2.2 to 3.9; aOR_women_: 1.3, 95% CI 0.8 to 2.0; table 3). Similarly, fall-related injuries and traumatic brain injuries were associated with a threefold risk of self-harm in men with depression (aOR range: 2.9–3.1, table 3) compared with a twofold risk increase in women with depression (aOR range: 1.9–2.0, table 3). There was some variation in age-specific associations, but the precision of the estimates was limited with wide CIs (online supplemental etable 3).

**Table 3:** aORs of self-harm in the week following exposure to any of the triggers (eg, fall-related injury, transport-related injury, traumatic brain injury and injury from interpersonal assault) compared with earlier unexposed periods within the same person, stratified by psychiatric diagnostic categories (eg, schizophrenia-spectrum disorder, bipolar disorder, depression and those without psychiatric disorders) and sex

	All individuals (reference)	Men	Women
	aOR (95% CI)	aOR (95% CI)	aOR (95% CI)
Schizophrenia-spectrum disorders			
Fall-related injury	2.1 (1.8 to 2.4)	2.4 (2.0 to 2.8)	1.9 (1.6 to 2.3)
Transport-related injury	2.7 (2.1 to 3.5)	3.3 (2.4 to 4.7)	2.1 (1.4 to 3.2)
Traumatic brain injury	2.4 (2.0 to 2.9)	2.7 (2.2 to 3.4)	2.1 (1.6 to 2.7)
Injury from interpersonal assault	2.0 (1.6 to 2.5)	2.9 (2.2 to 3.9)	1.3 (0.8 to 2.0)
Bipolar disorder			
Fall-related injury	2.0 (1.6 to 2.4)	2.7 (2.0 to 3.6)	1.7 (1.3 to 2.2)
Transport-related injury	2.9 (2.1 to 4.1)	3.5 (2.1 to 5.7)	2.7 (1.7 to 4.4)
Traumatic brain injury	2.9 (2.3 to 3.7)	2.7 (1.9 to 3.8)	3.1 (2.2 to 4.3)
Injury from interpersonal assault	2.7 (1.9 to 3.8)	3.4 (2.1 to 5.6)	2.4 (1.5 to 3.9)
Depression			
Fall-related injury	2.4 (2.1 to 2.6)	2.9 (2.5 to 3.4)	2.0 (1.8 to 2.4)
Transport-related injury	2.4 (1.9 to 2.9)	2.4 (1.8 to 3.2)	2.3 (1.8 to 3.1)
Traumatic brain injury	2.6 (2.3 to 3.0)	3.1 (2.7 to 3.7)	1.9 (1.5 to 2.4)
Injury from interpersonal assault	2.7 (2.3 to 3.3)	3.2 (2.5 to 4.1)	2.3 (1.8 to 3.0)
No psychiatric disorders			
Fall-related injury	6.9 (6.2 to 7.6)	6.5 (5.8 to 7.3)	7.7 (6.5 to 9.2)
Transport-related injury	6.3 (5.4 to 7.4)	6.1 (5.0 to 7.4)	6.2 (4.6 to 8.4)
Traumatic brain injury	7.3 (6.4 to 8.4)	7.5 (6.4 to 8.7)	7.1 (5.3 to 9.4)
Injury from interpersonal assault	7.6 (6.3 to 9.3)	7.8 (6.3 to 9.7)	7.1 (4.6 to 11.0)

Notes: The within-individual comparisons were adjusted for age, calendar month and all time-stable individual-level unmeasured confounders (eg, genetic risks, early childhood environmental factors and early-onset neurodevelopmental disorders). The psychiatric disorders were defined hierarchically.

aOR, adjusted OR.

In complementary sensitivity analyses, we did not find any material differences when we examined country-specific associations (online supplemental etable 4), allowed the control periods to randomly start up to 14 days later to account for potential within-month seasonality bias (online supplemental etable 5), used control periods that had occurred up to 3 years after the trigger events (online supplemental etable 6), restricted the control periods to 12 months (online supplemental etable 7), considered the first trigger event only (online supplemental etable 8), used narrower definitions of psychiatric disorders (online supplemental etable 9), considered suicidal behaviours as outcome (online supplemental etable 10), excluded trigger events in individuals who had engaged in self-harm within the previous 3 months (online supplemental etable 11) and restricted the cohorts to those who were 12 years old or younger when the self-harm data became available to account for left truncation bias (online supplemental etable 12). The associations were reduced in individuals without psychiatric disorders when self-harm events of undetermined intent were excluded (aORs: 2.8–3.9 vs 6.3–7.6) but not in those diagnosed with any of the psychiatric disorders (online supplemental etable 13).

## Discussion

In this population-based study of 249 210 individuals diagnosed with psychiatric disorders and 1 700 500 people without such diagnoses across Sweden and Finland, we examined the role of specific physical injuries as triggers for self-harm. We report three main findings.

First, we demonstrated that individuals with psychiatric disorders were approximately two to three times more likely to self-harm in the week following a physical injury compared with earlier control periods. By allowing these individuals to serve as their own controls over time, we were able to account for unmeasured and time-constant individual-level confounders (eg, genetics, early-life environmental risks and neurodevelopmental disorders). We did not find any consistent moderation effects across sex and age, and complementary sensitivity analyses showed the results were robust against different statistical model assumptions, moderator and outcome definitions, and potential reverse causation bias.

Second, although absolute risks of self-harm following these triggers were larger in people with schizophrenia-spectrum disorder and bipolar disorder (mean: 36.2 events per 10 000 person-weeks, range: 25.0–52.0) compared with depression (mean: 21.1 events per 10 000 person-weeks, range: 12.4–36.7), there were no clear differences between diagnostic categories in the relative risks after accounting for potential confounders.

Third, relative risks of the within-individual associations between triggers and self-harm were stronger in the general population without psychiatric disorders, conferring sixfold to eightfold relative risk increase in the week following the triggering injuries compared with themselves during earlier control periods. These findings should, however, be interpreted in light of very low absolute baseline risks of self-harm in the general population compared with individuals with psychiatric disorders (≤1.2 events vs averaging 11.5 events per 10 000 person-weeks). These findings are broadly consistent with earlier reports that have either estimated larger relative risks in people without rather than with psychiatric disorders^12^ or have lacked sufficient statistical power to identify such differences.^16^

Our findings are consistent with a growing body of literature that has found specific physical injuries, including head injuries^17^ and violent victimisation occurring either in intimate partner^18 19^ or prison settings,^20^ to act as distal risk factors for suicidal behaviours. The present study adds to these findings by identifying four types of physical injuries (ie, fall-related injuries, transport-related injuries, traumatic brain injuries and injuries from interpersonal assault) as proximal risk factors or triggers for self-harm, with the strength of the associations varying across people with distinct psychiatric disorders and the general population. However, more are needed to understand the mechanisms involved. Research can examine the effect on brain function using neuroimaging, neurochemical and neuropsychological approaches to investigate possible pathways. The role of axonal injuries, disruptions to serotonergic and noradrenergic, and temporary impairments to decision making have been proposed.^21^ In terms of clinical implications, our findings underscore the necessity of developing effective interventions that prevent physical injuries in people with psychiatric disorders, as such injuries may potentially have far-reaching behavioural consequences.^14^ Potential targets for such interventions may include reducing feelings of hopelessness and related depressive symptoms, irritability, impulsivity, comorbid substance misuse and medication non-adherence. Emergency and trauma-based medical services should consider self-harm risk and stronger liaison with psychiatric services. Following a physical injury, psychiatric services should conduct evidence-based risk assessments of self-harm in patients with psychiatric disorders to inform more frequent and intensive follow-up and underscore safety planning.

Strengths of the study included the use of two nationwide registry datasets that enabled us to study over 1.9 million people who had at least one physical injury requiring medical care, stratified by the presence of diagnosed psychiatric disorders. This approach further enabled us to keep selection and attrition biases to a minimum as both countries offer their residents universal healthcare coverage, the services of which having been carefully documented in national registries as mandated by law. The large sample size and research design allowed us to assess physical injuries as triggers for self-harm within specific diagnostic categories of psychiatric disorders while accounting for unmeasured individual-level confounders and without self-report biases.

A number of limitations should be noted. First, an unknown proportion of the physical injury exposures may be misclassified self-harm events. For non-fatal car accidents in the general population, it has been conservatively estimated that at least 2% of hospital patients who had been injured in car accidents may have attempted suicide using a car.^22^ Less is known about this type of misclassification bias in other injury types and any potential differences between the general population and patients with psychiatric disorders. However, we found very stable results across all four types of physical injuries that we examined (including those experiencing victimisation for whom misclassified self-harm biases are likely negligible), and our complementary sensitivity analyses did not suggest that reverse causation bias had any material impact on the findings. Importantly, our results are further aligned with a Swedish study^23^ that examined bereavement of close relatives as proximal risk factors of suicide, which does not suffer from this misclassification bias and highlights the importance of trauma as an important mechanism contributing to elevated risks of suicidal behaviours. The presence of this type of misclassification bias could potentially result in artificially inflated associations between the triggers and self-harm. In our complementary sensitivity analyses, we found that excluding self-harm events of undetermined intent substantially reduced the associations between the triggers and self-harm, but only in individuals without psychiatric disorders. The latter findings suggest that those without psychiatric disorders may be more susceptible to this type of misclassification bias than those with psychiatric disorders. Second, despite being able to account for time-stable unmeasured confounders, our estimates could be confounded by time-varying confounders. We did not account for time-varying sociodemographic confounders because nationwide population registries typically gather annual measures of such indicators, which rarely impact associations measured in shorter time intervals.^14 24^ Furthermore, in complementary sensitivity analyses, we did not find that restricting the follow-up period to 12 months to reduce the likelihood of large shifts in the socioeconomic status of the participants changed our findings. While residual confounding may account for a portion of our findings, we note that even if such factors account for approximately half of the estimated within-individual associations, the associations would remain large. Third, we were unable to determine whether the distinct physical injury diagnoses, including self-harm, were made by the same or different physicians. Despite the lack of specific data for emergency medicine and psychiatry, we note that only about one-third of patients in Finland and Sweden report having a stable clinical contact with a physician or nurse, compared with over 80% in many other high-income nations.^25 26^ Fourth, we used a hierarchical definition of psychiatric disorders based on externally validated diagnoses of schizophrenia-spectrum disorder, bipolar disorder and depression. Due to the nature of patient registers, however, we were unable to obtain accurate temporal data on the onset of these conditions and self-harm, as we only had access to hospital presentations for each individual. Future studies may benefit from using richer clinical datasets to examine aetiological links between physical injuries and self-harm throughout the life course, including relative contributions of psychiatric symptoms, impulsivity, cognitions and related measures, for which we did not have reliable data. Possible moderating effects of the contexts in which physical injuries occur (eg, falls in the workplace vs at home or automobile accidents involving one or multiple vehicles) could also be investigated.

Two sets of findings support the generalisability of our findings. First, our complementary analyses indicated that the country-specific estimates for the associations between the triggers and self-harm were not meaningfully different from each other. Second, age-standardised rates of falls,^27^ road injuries,^28^ traumatic brain injuries^29^ and self-harm^2^ have been found to be similar across Sweden, Finland and other countries with a high sociodemographic index score. Similarly, self-reported violent victimisation prevalence rates measured in Finland (2.2%) and Sweden (3.5%) were similar to the global average (3.1%) between 2003 and 2004.^30^

## Conclusions

In this study of physical injuries as proximal risk factors for self-harm across two countries, in those with psychiatric disorders, we found that risks of self-harm were elevated by two to three times in the week following a physical injury compared with earlier unexposed periods. By using each individual as their own controls, we were able to account for stable individual-level confounders, including genetic and early environmental risks. Absolute risks of self-harm were considerably elevated in people with psychiatric disorders than in the general population without psychiatric disorders. The findings highlight the importance of combining injury prevention strategies to reduce self-harm at both targeted and general population levels. They also indicate the importance of including inquiry about physical injuries in assessment of people who self-harm, both to assess their role in the aetiology of the behaviour and to indicate issues which may require specific therapeutic interventions.

## Data Availability

Data may be obtained from a third party and are not publicly available. Finnish and Swedish privacy laws prohibit us from making individual-level data publicly available. Researchers who are interested in replicating our work using individual-level data can seek access via Findata, Statistics Sweden and the Swedish National Board for Health and Welfare. For more information, see https://findata.fi/en/ and https://www.scb.se/en/services/ordering-data-and-statistics/ordering-microdata/.
