# Supplementary material for: Physical injuries as triggers for self-harm: a within-individual study of nearly 250 000 injured people with a major psychiatric disorder
Source: BMJ Ment Health. 2023 Jun 28;26(1):e300758. doi: 10.1136/bmjment-2023-300758 (PMC10577735; doi:10.1136/bmjment-2023-300758)
Supplement: Supplementary data [file bmjment-2023-300758supp001.pdf]

eText 1. Validity of diagnoses of psychiatric disorders and physical injuries

Reviews of the inpatient registers in both countries have reported excellent validity with positive predictive values (PPVs) for most diagnoses ranging between 85-95% in Sweden and 75-99% in Finland.[1,2] Validity studies of single-episode diagnoses have demonstrated excellent validity for schizophrenia spectrum disorders (Sweden: ϰ=0.86;[3] Finland: PPV≥0.84[2]) and bipolar disorder (Sweden: concordance rate of 86% compared to file reviews by psychiatrists[4]; Finland: PPV range between 87-93%[5]). The validity of single-episode depression diagnoses has similarly been shown to be fair to moderate in Sweden when tested against clinical forensic registers (ϰ=0.31, 88% full agreement)[6] and good in Finland when compared to researcher assessments (ϰ=0.65, PPV=0.81).[7] Individuals in the general population were defined as those who had never been diagnosed with a psychiatric disorder, with the exception of substance use disorder.

Although broader measures of injury-related diagnoses have been found to have excellent validity in both Sweden and in Finland (PPVs>90%),[1,2] we are unaware of any studies that have focused on specific injury types.

References

1 Ludvigsson J, Andersson E, Ekbom A, *et al.* External review and validation of the Swedish national inpatient register. *BMC Public Health* 2011; **11**: 450.

2 Sund R. Quality of the Finnish Hospital Discharge Register: A systematic review. *Scandinavian Journal of Public Health* 2012; **40**: 505–15.

3 Fazel S, Långström N, Hjern A, Grann M, Lichtenstein P. Schizophrenia, substance abuse, and violent crime. *JAMA* 2009; **301**: 2016.

4 Sellgren C, Landén M, Lichtenstein P, Hultman CM, Långström N. Validity of bipolar disorder hospital discharge diagnoses: file review and multiple register linkage in Sweden. *Acta Psychiatrica Scandinavica* 2011; **124**: 447–53.

5 Kieseppä T, Partonen T, Kaprio J, Lönnqvist J. Accuracy of register- and record-based bipolar I disorder diagnoses in Finland; a study of twins. *Acta Neuropsychiatr* 2000; **12**: 106–9.

6 Fazel S, Wolf A, Chang Z, Larsson H, Goodwin GM, Lichtenstein P. Depression and violence: a Swedish population study. *Lancet Psychiatry* 2015; **2**: 224–32.

7 Taiminen T, Ranta K, Karlsson H, *et al.* Comparison of clinical and best-estimate research DSM-IV diagnoses in a Finnish sample of first-admission psychosis and severe affective disorder. *Nordic Journal of Psychiatry* 2001; **55**: 107–11.

eText 2. Complementary sensitivity analyses

First, to test for the generalizability of the findings across both countries, we compared the pooled estimates with equivalent country-specific estimates (**eTable 4**). To test for potential within-month seasonality bias, we re-ran the analyses in a new sample where the start dates of the 7-day control periods were randomly moved forward between 0 and 14 days (**eTable 5**). Similarly, we tested for the potential bias of selecting control periods that had occurred prior to the trigger exposure by considering equivalent control periods that had occurred up to three years after the trigger event (**eTable 6**). We further considered the impact of reducing the number of control periods down to 12 months (**eTable 7**) and to only consider the first trigger event (**eTable 8**). We also tested for alternative definitions of the psychiatric disorders (e.g., at least two diagnoses on separate occasions and excluding diagnoses being given in outpatient care settings; **eTable 9**) and outcome (i.e., suicidal behaviours; **eTable 10**). We further tested for the impact of reverse causation bias by removing trigger and control periods that had been preceded by self-harm in the last three months (**eTable 11**) and left-truncation bias by restricting the cohorts to those who were 12 years old or younger when the self-harm data were made available (Finland: 1975-2000; Sweden: 1961-1993; **eTable 12**). Lastly, we tested whether excluding self-harm events of undetermined intent altered our findings (**eTable 13**).

eTable 1. ICD codes

	ICD-8	ICD-9	ICD-10
**Any psychiatric disorder**	290-315	290-319	F00-F99
**Substance use disorder (used as an exclusion criteria)**			
Finland	291, 303, 304	291, 292, 303, 304, 305 [excl. *.1]	F10-F12, F14-F16, F19 [excl. F1*.5]
Sweden	291, 303, 304	291, 292, 303, 304, 305A, 305X	F10-F12, F14-F16, F19 [excl. F1*.5]
**Schizophrenia-spectrum disorder**	295, 297-299	295, 297-298	F20-F29
**Bipolar disorder**			
Finland	296 [excl. 296.2]	296 [excl. 296.1A-D, 296.1F-G]	F30-F31
Sweden	296 [excl. 296.2]	296 [excl. 296B]	F30-F31
**Depression**			
Finland	296.2, 300.4	296.1 [excl. 296.1E], 296.8A, 300.4A	F32-F39 [excl. F32.3 and F33.3]
Sweden	296.2, 300.4	296B, 300E, 311	F32-F39 [excl. F32.3 and F33.3]
**Fall-related injury**			
Finland	-	E880-E889	W00-W19
Sweden	E880-E887	E880-E888	W00-W19

**Transport-related injury**			
Finland	-	E800-E830A	V01-V99
Sweden	E807-E846	E800-E849	V01-V99
**Traumatic brain injury**	800-804, 850–854	800-804, 850–854	S01.0–S01.9, S02.0, S02.1, S02.3, S02.7–S02.9, S04.0, S06.0–S06.9, S07.0, S07.1, S07.8. S07.9, S09.7–S09.9, T01.0, T02.0, T04.0, T06.0, T90.1, T90.2, T90.4, T90.5, T90.8, T90.9
**Injury from interpersonal assault**	E960-E969	E960-E969	X85-X99, Y00-Y09
**Suicidal behaviour (self-harm and/or suicide)**	851–854	851–854	
Finland	-	E950A-E959X, E970A-E979A	X60-X84, Y10-Y34
Sweden	E950-E959, E980-E989	E950-E959, E980-E989	X60-X84, Y10-Y34
**Self-harm of undetermined intent (used as an exclusion criteria)**			
Finland	-	E970A-E979A	Y10-Y34
Sweden	E980-E989	E980-E989	Y10-Y34

eTable 2. Number of observations in person-weeks and rates of suicidal behaviours per 10,000 person-weeks across all periods, control periods and periods following each trigger

	Schizophrenia-spectrum disorder		Bipolar disorder		Depression		No psychiatric disorders	
	**Observations**	**Rate [95% CI]**	**Observations**	**Rate [95% CI]**	**Observations**	**Rate [95% CI]**	**Observations**	**Rate [95% CI]**
Fall-related injury								
All periods	1,788,010	14.4 [13.9; 15.0]	1,028,294	11.7 [11.0; 12.4]	6,145,348	6.1 [5.9; 6.3]	54,255,361	0.5 [0.5; 0.5]
Control periods	1,709,463	13.5 [12.9; 14.0]	983,923	11.1 [10.4; 11.8]	5,875,476	5.7 [5.5; 5.9]	52,124,783	0.4 [0.4; 0.4]
Following trigger	78,547	34.8 [30.8; 39.1]	44,371	25.0 [20.6; 30.1]	269,872	15.3 [13.8; 16.8]	2,130,578	2.7 [2.4; 2.9]
Transport-related injury								
All periods	616,219	10.4 [9.6; 11.2]	382,319	10.0 [9.0; 11.0]	2,305,366	5.3 [5.0; 5.6]	19,752,339	0.5 [0.5; 0.5]
Control periods	591,214	9.7 [8.9; 10.5]	366,746	9.2 [8.3; 10.3]	2,206,143	5.0 [4.7; 5.3]	19,022,005	0.4 [0.4; 0.4]
Following trigger	25,005	26.8 [20.8; 34.0]	15,573	27.6 [20.0; 37.2]	99,223	12.4 [10.3; 14.8]	730,334	2.8 [2.4; 3.2]
Traumatic brain injury								
All periods	1,073,411	14.0 [13.3; 14.8]	558,967	13.3 [12.4; 14.3]	3,128,254	7.5 [7.2; 7.9]	20,323,253	0.7 [0.6; 0.7]
Control periods	1,032,080	13.1 [12.4; 13.8]	538,776	12.4 [11.5; 13.4]	3,005,817	7.0 [6.7; 7.3]	19,666,398	0.5 [0.5; 0.6]
Following trigger	41,331	37.5 [31.8; 43.9]	20,191	37.6 [29.7; 47.1]	122,437	19.8 [17.4; 22.5]	656,855	4.4 [3.9; 5.0]
Injury from interpersonal assault								
All periods	439,375	21.2 [19.9; 22.6]	218,099	19.8 [17.9; 21.7]	1,136,691	12.8 [12.1; 13.4]	4,279,902	1.5 [1.4; 1.6]
Control periods	423,646	20.2 [18.9; 21.6]	210,596	18.6 [16.8; 20.5]	1,096,598	11.9 [11.3; 12.6]	4,147,447	1.2 [1.1; 1.3]
Following trigger	15,729	48.3 [38.1; 60.4]	7503	52.0 [37.0; 71.0]	40,093	36.7 [31.0; 43.1]	132,455	11.1 [9.4; 13.0]

Notes: The psychiatric disorders were defined hierarchically.

eTable 3. Sensitivity test: Age-specific estimates

	All ages (reference)	Ages 12-19 years	Ages 20-29 years	Ages 30-39 years	Ages 40-49 years	Ages 50 years or older
	**aOR [95% CI]**	**aOR [95% CI]**	**aOR [95% CI]**	**aOR [95% CI]**	**aOR [95% CI]**	**aOR [95% CI]**
**Schizophrenia-spectrum disorders**						
Fall-related injury	2.1 [1.8; 2.4]	1.5 [0.8; 2.6]	2.0 [1.6; 2.5]	2.2 [1.7; 2.8]	1.7 [1.3; 2.3]	1.6 [1.0; 2.4]
Transport-related injury	2.7 [2.1; 3.5]	1.0 [0.3; 3.7]	2.5 [1.6; 3.9]	2.1 [1.2; 3.7]	3.2 [1.6; 6.3]	14.6 [4.7; 45.2]
Traumatic brain injury	2.4 [2.0; 2.9]	1.0 [0.4; 2.3]	2.3 [1.7; 3.0]	2.2 [1.6; 3.1]	1.8 [1.2; 2.8]	3.9 [2.2; 6.9]
Injury from interpersonal assault	2.0 [1.6; 2.5]	1.7 [0.6; 4.6]	1.2 [0.8; 1.9]	2.1 [1.3; 3.3]	2.4 [1.4; 4.0]	2.3 [0.8; 6.8]
**Bipolar disorder**						
Fall-related injury	2.0 [1.6; 2.4]	1.1 [0.5; 2.3]	2.3 [1.6; 3.3]	1.8 [1.1; 2.9]	1.6 [1.1; 2.5]	1.8 [1.0; 2.9]
Transport-related injury	2.9 [2.1; 4.1]	3.8 [1.2; 11.4]	2.2 [1.1; 4.5]	3.0 [1.6; 5.8]	4.3 [2.3; 8.1]	1.6 [0.2; 16.5]
Traumatic brain injury	2.9 [2.3; 3.7]	3.0 [1.3; 6.9]	2.9 [1.8; 4.7]	3.5 [2.2; 5.5]	3.0 [1.8; 5.0]	0.8 [0.3; 2.4]
Injury from interpersonal assault	2.7 [1.9; 3.8]	1.6 [0.3; 8.8]	2.3 [1.1; 4.8]	3.6 [2.0; 6.4]	3.6 [1.7; 7.5]	3.9 [0.4; 39.6]
**Depression**						
Fall-related injury	2.4 [2.1; 2.6]	2.5 [1.8; 3.4]	2.2 [1.8; 2.7]	2.3 [1.8; 2.9]	2.0 [1.6; 2.5]	2.0 [1.5; 2.6]
Transport-related injury	2.4 [1.9; 2.9]	1.3 [0.7; 2.1]	1.9 [1.3; 2.7]	3.0 [2.0; 4.7]	2.8 [1.7; 4.4]	1.8 [0.8; 3.7]
Traumatic brain injury	2.6 [2.3; 3.0]	1.5 [0.9; 2.4]	2.1 [1.6; 2.7]	2.4 [1.8; 3.2]	2.6 [1.9; 3.5]	3.6 [2.4; 5.3]
Injury from interpersonal assault	2.7 [2.3; 3.3]	1.9 [1.1; 3.2]	2.0 [1.4; 2.7]	3.3 [2.4; 4.7]	2.6 [1.6; 4.1]	3.2 [1.6; 6.5]
**No psychiatric disorders**						
Fall-related injury	6.9 [6.2; 7.6]	5.1 [4.0; 6.5]	5.6 [4.6; 6.8]	5.2 [4.0; 6.7]	8.1 [6.4; 10.3]	6.2 [4.8; 8.0]
Transport-related injury	6.3 [5.4; 7.4]	6.0 [4.3; 8.5]	4.5 [3.2; 6.2]	6.9 [4.4; 10.6]	5.3 [3.2; 8.6]	7.9 [4.5; 13.6]
Traumatic brain injury	7.3 [6.4; 8.4]	5.2 [3.6; 7.4]	5.5 [4.2; 7.2]	7.7 [5.6; 10.8]	7.7 [5.5; 10.8]	9.8 [6.3; 15.1]
Injury from interpersonal assault	7.6 [6.3; 9.3]	8.5 [5.2; 13.9]	7.2 [5.0; 10.3]	4.4 [2.6; 7.2]	11.5 [6.7; 19.7]	4.5 [1.7; 11.6]

Notes: The within-individual comparisons were adjusted for age, calendar month and all time-stable individual-level unmeasured confounders (e.g., genetic risks, early childhood environmental factors, and early-onset neurodevelopmental disorders). The psychiatric disorders were defined hierarchically.

eTable 4. Sensitivity test: Country-specific estimates

	Pooled (reference)	Finland	Sweden
	aOR [95% CI]	aOR [95% CI]	aOR [95% CI]
**Schizophrenia-spectrum disorders**			
Fall-related injury	2.1 [1.8; 2.4]	1.9 [1.5; 2.3]	2.3 [1.9; 2.7]
Transport-related injury	2.7 [2.1; 3.5]	2.4 [1.6; 3.5]	2.9 [2.0; 4.2]
Traumatic brain injury	2.4 [2.0; 2.9]	2.6 [2.1; 3.3]	2.2 [1.7; 2.9]
Injury from interpersonal assault	2.0 [1.6; 2.5]	2.3 [1.6; 3.1]	1.7 [1.2; 2.5]
**Bipolar disorder**			
Fall-related injury	2.0 [1.6; 2.4]	2.3 [1.7; 3.1]	1.8 [1.4; 2.4]
Transport-related injury	2.9 [2.1; 4.1]	3.1 [1.9; 5.2]	2.8 [1.8; 4.4]
Traumatic brain injury	2.9 [2.3; 3.7]	2.6 [1.8; 3.7]	3.2 [2.3; 4.4]
Injury from interpersonal assault	2.7 [1.9; 3.8]	2.5 [1.5; 4.2]	2.9 [1.9; 4.5]
**Depression**			
Fall-related injury	2.4 [2.1; 2.6]	2.5 [2.1; 2.9]	2.3 [2.0; 2.7]
Transport-related injury	2.4 [1.9; 2.9]	2.2 [1.6; 3.1]	2.4 [1.9; 3.1]
Traumatic brain injury	2.6 [2.3; 3.0]	2.8 [2.3; 3.4]	2.4 [2.0; 2.9]
Injury from interpersonal assault	2.7 [2.3; 3.3]	2.9 [2.2; 3.9]	2.6 [2.1; 3.3]
**No psychiatric disorders**			
Fall-related injury	6.9 [6.2; 7.6]	4.5 [3.5; 5.8]	7.4 [6.7; 8.2]
Transport-related injury	6.3 [5.4; 7.4]	4.8 [3.1; 7.4]	6.6 [5.5; 7.9]
Traumatic brain injury	7.3 [6.4; 8.4]	6.3 [4.7; 8.4]	7.6 [6.5; 8.9]
Injury from interpersonal assault	7.6 [6.3; 9.3]	8.7 [6.2; 12.3]	7.2 [5.6; 9.1]

Notes: The within-individual comparisons were adjusted for age, calendar month and all time-stable individual-level unmeasured confounders (e.g., genetic risks, early childhood environmental factors, and early-onset neurodevelopmental disorders). The psychiatric disorders were defined hierarchically.

eTable 5. Sensitivity test: Each control period for each individual was randomly moved forward for a period of between 0 and 14 days to test for potential within-month seasonality bias

	**Main analysis (reference)**	**Random start dates for the control periods**
	**aOR [95% CI]**	**aOR [95% CI]**
**Schizophrenia-spectrum disorders**		
Fall-related injury	2.1 [1.8; 2.4]	2.1 [1.8; 2.3]
Transport-related injury	2.7 [2.1; 3.5]	2.9 [2.2; 3.8]
Traumatic brain injury	2.4 [2.0; 2.9]	2.4 [2.1; 2.9]
Injury from interpersonal assault	2.0 [1.6; 2.5]	2.1 [1.7; 2.7]
**Bipolar disorder**		
Fall-related injury	2.0 [1.6; 2.4]	2.0 [1.6; 2.4]
Transport-related injury	2.9 [2.1; 4.1]	2.9 [2.1; 4.1]
Traumatic brain injury	2.9 [2.3; 3.7]	2.9 [2.3; 3.7]
Injury from interpersonal assault	2.7 [1.9; 3.8]	2.6 [1.9; 3.7]
**Depression**		
Fall-related injury	2.4 [2.1; 2.6]	2.3 [2.1; 2.6]
Transport-related injury	2.4 [1.9; 2.9]	2.4 [1.9; 2.9]
Traumatic brain injury	2.6 [2.3; 3.0]	2.5 [2.2; 2.9]
Injury from interpersonal assault	2.7 [2.3; 3.3]	2.5 [2.1; 3.0]
**No psychiatric disorders**		
Fall-related injury	6.9 [6.2; 7.6]	6.5 [5.9; 7.1]
Transport-related injury	6.3 [5.4; 7.4]	6.4 [5.4; 7.5]
Traumatic brain injury	7.3 [6.4; 8.4]	7.8 [6.8; 8.9]
Injury from interpersonal assault	7.6 [6.3; 9.3]	8.0 [6.6; 9.8]

Notes: The within-individual comparisons were adjusted for age, calendar month and all time-stable individual-level unmeasured confounders (e.g., genetic risks, early childhood environmental factors, and early-onset neurodevelopmental disorders). The psychiatric disorders were defined hierarchically.

eTable 6. Adjusted odds ratios of self-harm in the week following exposure to any of the triggers (e.g., fall-related accident, transport-related accident, traumatic brain injury, and violent victimisation) compared to later unexposed periods within the same person, stratified by psychiatric diagnostic categories (e.g., schizophrenia-spectrum disorder, bipolar disorder, depression, and those without psychiatric disorders)

	Control periods that occurred up to three years before the trigger event (reference)	Control periods that occurred up to three years after the trigger event
	**aOR [95% CI]**	**aOR [95% CI]**
**Schizophrenia-spectrum disorders**		
Fall-related injury	2.1 [1.8; 2.4]	2.2 [1.8; 2.8]
Transport-related injury	2.7 [2.1; 3.5]	2.8 [2.1; 3.8]
Traumatic brain injury	2.4 [2.0; 2.9]	2.7 [2.1; 3.4]
Injury from interpersonal assault	2.0 [1.6; 2.5]	2.6 [2.0; 3.5]
**Bipolar disorder**		
Fall-related injury	2.0 [1.6; 2.4]	2.7 [2.1; 3.6]
Transport-related injury	2.9 [2.1; 4.1]	3.5 [2.3; 5.4]
Traumatic brain injury	2.9 [2.3; 3.7]	2.9 [2.1; 3.9]
Injury from interpersonal assault	2.7 [1.9; 3.8]	2.5 [1.7; 3.9]
**Depression**		
Fall-related injury	2.4 [2.1; 2.6]	3.0 [2.6; 3.5]
Transport-related injury	2.4 [1.9; 2.9]	2.6 [2.1; 3.3]
Traumatic brain injury	2.6 [2.3; 3.0]	2.6 [2.1; 3.1]
Injury from interpersonal assault	2.7 [2.3; 3.3]	2.6 [2.1; 3.3]
**No psychiatric disorders**		
Fall-related injury	6.9 [6.2; 7.6]	6.7 [6.0; 7.6]
Transport-related injury	6.3 [5.4; 7.4]	4.3 [3.5; 5.3]
Traumatic brain injury	7.3 [6.4; 8.4]	6.2 [5.2; 7.3]
Injury from interpersonal assault	7.6 [6.3; 9.3]	7.6 [6.0; 9.5]

Notes: The within-individual comparisons were adjusted for age, calendar month and all time-stable individual-level unmeasured confounders (e.g., genetic risks, early childhood environmental factors, and early-onset neurodevelopmental disorders). The psychiatric disorders were defined hierarchically.

eTable 7. Sensitivity test: Limiting the control periods to 12 months instead of 36 months per person.

	36 months (reference)	12 months
	**aOR [95% CI]**	**aOR [95% CI]**
**Schizophrenia-spectrum disorders**		
Fall-related injury	2.1 [1.8; 2.4]	1.8 [1.6; 2.0]
Transport-related injury	2.7 [2.1; 3.5]	2.5 [1.9; 3.4]
Traumatic brain injury	2.4 [2.0; 2.9]	2.1 [1.7; 2.5]
Injury from interpersonal assault	2.0 [1.6; 2.5]	1.7 [1.3; 2.2]
**Bipolar disorder**		
Fall-related injury	2.0 [1.6; 2.4]	1.8 [1.5; 2.2]
Transport-related injury	2.9 [2.1; 4.1]	2.6 [1.8; 3.7]
Traumatic brain injury	2.9 [2.3; 3.7]	2.5 [1.9; 3.2]
Injury from interpersonal assault	2.7 [1.9; 3.8]	2.2 [1.5; 3.1]
**Depression**		
Fall-related injury	2.4 [2.1; 2.6]	2.1 [1.8; 2.3]
Transport-related injury	2.4 [1.9; 2.9]	2.0 [1.6; 2.4]
Traumatic brain injury	2.6 [2.3; 3.0]	2.3 [2.0; 2.6]
Injury from interpersonal assault	2.7 [2.3; 3.3]	2.4 [2.0; 2.9]
**No psychiatric disorders**		
Fall-related injury	6.9 [6.2; 7.6]	5.5 [5.0; 6.1]
Transport-related injury	6.3 [5.4; 7.4]	5.2 [4.4; 6.2]
Traumatic brain injury	7.3 [6.4; 8.4]	6.3 [5.4; 7.3]
Injury from interpersonal assault	7.6 [6.3; 9.3]	6.5 [5.3; 8.1]

Notes: The within-individual comparisons were adjusted for age, calendar month and all time-stable individual-level unmeasured confounders (e.g., genetic risks, early childhood environmental factors, and early-onset neurodevelopmental disorders). The psychiatric disorders were defined hierarchically.

eTable 8. Sensitivity test: Limiting the analyses to the first trigger event instead of all trigger events throughout the follow-up period

	All trigger events (reference)	First trigger event
	aOR [95% CI]	aOR [95% CI]
**Schizophrenia-spectrum disorders**		
Fall-related injury	2.1 [1.8; 2.4]	2.0 [1.6; 2.5]
Transport-related injury	2.7 [2.1; 3.5]	3.1 [2.2; 4.4]
Traumatic brain injury	2.4 [2.0; 2.9]	2.4 [1.9; 3.2]
Injury from interpersonal assault	2.0 [1.6; 2.5]	2.0 [1.5; 2.8]
**Bipolar disorder**		
Fall-related injury	2.0 [1.6; 2.4]	2.0 [1.5; 2.7]
Transport-related injury	2.9 [2.1; 4.1]	2.8 [1.8; 4.4]
Traumatic brain injury	2.9 [2.3; 3.7]	2.6 [1.8; 3.8]
Injury from interpersonal assault	2.7 [1.9; 3.8]	1.7 [1.1; 2.8]
**Depression**		
Fall-related injury	2.4 [2.1; 2.6]	2.7 [2.3; 3.1]
Transport-related injury	2.4 [1.9; 2.9]	2.3 [1.8; 3.0]
Traumatic brain injury	2.6 [2.3; 3.0]	2.5 [2.1; 3.1]
Injury from interpersonal assault	2.7 [2.3; 3.3]	2.2 [1.7; 2.8]
**No psychiatric disorders**		
Fall-related injury	6.9 [6.2; 7.6]	7.4 [6.5; 8.6]
Transport-related injury	6.3 [5.4; 7.4]	6.7 [5.3; 8.3]
Traumatic brain injury	7.3 [6.4; 8.4]	7.8 [6.4; 9.4]
Injury from interpersonal assault	7.6 [6.3; 9.3]	7.0 [5.4; 9.1]

Notes: The within-individual comparisons were adjusted for age, calendar month and all time-stable individual-level unmeasured confounders (e.g., genetic risks, early childhood environmental factors, and early-onset neurodevelopmental disorders). The psychiatric disorders were defined hierarchically.

eTable 9. Sensitivity test: Restricting the definitions of the psychiatric disorders to patients who were diagnosed with the conditions at two separate occasions or those who were only diagnosed in inpatient care settings

	Main analysis (reference)	At least 2 diagnoses on separate occasions	Inpatient care only
	**aOR [95% CI]**	**aOR [95% CI]**	**aOR [95% CI]**
**Schizophrenia-spectrum disorders**			
Fall-related injury	2.1 [1.8; 2.4]	2.2 [1.9; 2.5]	2.1 [1.8; 2.5]
Transport-related injury	2.7 [2.1; 3.5]	2.7 [2.0; 3.7]	2.7 [2.0; 3.6]
Traumatic brain injury	2.4 [2.0; 2.9]	2.7 [2.2; 3.2]	2.4 [2.0; 2.9]
Injury from interpersonal assault	2.0 [1.6; 2.5]	2.0 [1.5; 2.7]	1.9 [1.5; 2.5]
**Bipolar disorder**			
Fall-related injury	2.0 [1.6; 2.4]	1.9 [1.5; 2.4]	1.9 [1.5; 2.3]
Transport-related injury	2.9 [2.1; 4.1]	2.7 [1.8; 3.9]	3.1 [2.1; 4.6]
Traumatic brain injury	2.9 [2.3; 3.7]	2.6 [2.0; 3.5]	2.7 [2.1; 3.6]
Injury from interpersonal assault	2.7 [1.9; 3.8]	2.4 [1.6; 3.5]	2.8 [1.9; 4.2]
**Depression**			
Fall-related injury	2.4 [2.1; 2.6]	2.3 [2.1; 2.6]	2.3 [2.0; 2.5]
Transport-related injury	2.4 [1.9; 2.9]	2.5 [2.1; 3.1]	2.1 [1.7; 2.7]
Traumatic brain injury	2.6 [2.3; 3.0]	2.4 [2.0; 2.7]	2.5 [2.2; 3.0]
Injury from interpersonal assault	2.7 [2.3; 3.3]	2.4 [2.0; 3.0]	2.5 [2.1; 3.1]

Notes: The within-individual comparisons were adjusted for age, calendar month and all time-stable individual-level unmeasured confounders (e.g., genetic risks, early childhood environmental factors, and early-onset neurodevelopmental disorders). The psychiatric disorders were defined hierarchically.

eTable 10. Sensitivity test: Examining the alternative outcome measure of suicidal behaviours (i.e., either self-harm or completed suicide)

	Self-harm (reference)	Suicidal behaviors
	**aOR [95% CI]**	**aOR [95% CI]**
**Schizophrenia-spectrum disorders**		
Fall-related injury	2.1 [1.8; 2.4]	2.1 [1.8; 2.4]
Transport-related injury	2.7 [2.1; 3.5]	2.7 [2.1; 3.5]
Traumatic brain injury	2.4 [2.0; 2.9]	2.4 [2.1; 2.9]
Injury from interpersonal assault	2.0 [1.6; 2.5]	2.0 [1.6; 2.5]
**Bipolar disorder**		
Fall-related injury	2.0 [1.6; 2.4]	2.0 [1.7; 2.5]
Transport-related injury	2.9 [2.1; 4.1]	3.1 [2.2; 4.3]
Traumatic brain injury	2.9 [2.3; 3.7]	3.0 [2.4; 3.8]
Injury from interpersonal assault	2.7 [1.9; 3.8]	2.7 [1.9; 3.8]
**Depression**		
Fall-related injury	2.4 [2.1; 2.6]	2.4 [2.2; 2.7]
Transport-related injury	2.4 [1.9; 2.9]	2.4 [2.0; 3.0]
Traumatic brain injury	2.6 [2.3; 3.0]	2.6 [2.3; 3.0]
Injury from interpersonal assault	2.7 [2.3; 3.3]	2.7 [2.3; 3.3]
**No psychiatric disorders**		
Fall-related injury	6.9 [6.2; 7.6]	7.1 [6.5; 7.8]
Transport-related injury	6.3 [5.4; 7.4]	6.5 [5.5; 7.6]
Traumatic brain injury	7.3 [6.4; 8.4]	7.6 [6.7; 8.7]
Injury from interpersonal assault	7.6 [6.3; 9.3]	7.7 [6.4; 9.4]

Notes: The within-individual comparisons were adjusted for age, calendar month and all time-stable individual-level unmeasured confounders (e.g., genetic risks, early childhood environmental factors, and early-onset neurodevelopmental disorders). The psychiatric disorders were defined hierarchically.

eTable 11. Sensitivity test: Testing for reverse causation bias by excluding trigger events that were preceded by self-harm up to three months earlier

	All triggers (reference)	Excluding triggers that were preceded by self-harm up to 3 months earlier
	**aOR [95% CI]**	**aOR [95% CI]**
**Schizophrenia-spectrum disorders**		
Fall-related injury	2.1 [1.8; 2.4]	2.4 [2.1; 2.8]
Transport-related injury	2.7 [2.1; 3.5]	3.6 [2.7; 4.7]
Traumatic brain injury	2.4 [2.0; 2.9]	3.1 [2.6; 3.8]
Injury from interpersonal assault	2.0 [1.6; 2.5]	2.5 [1.9; 3.3]
**Bipolar disorder**		
Fall-related injury	2.0 [1.6; 2.4]	2.3 [1.9; 2.9]
Transport-related injury	2.9 [2.1; 4.1]	3.5 [2.4; 5.1]
Traumatic brain injury	2.9 [2.3; 3.7]	3.5 [2.7; 4.5]
Injury from interpersonal assault	2.7 [1.9; 3.8]	3.3 [2.3; 4.8]
**Depression**		
Fall-related injury	2.4 [2.1; 2.6]	2.7 [2.4; 3.1]
Transport-related injury	2.4 [1.9; 2.9]	2.8 [2.3; 3.5]
Traumatic brain injury	2.6 [2.3; 3.0]	3.2 [2.7; 3.7]
Injury from interpersonal assault	2.7 [2.3; 3.3]	3.2 [2.6; 3.9]
**No psychiatric disorders**		
Fall-related injury	6.9 [6.2; 7.6]	8.2 [7.4; 9.1]
Transport-related injury	6.3 [5.4; 7.4]	7.8 [6.6; 9.2]
Traumatic brain injury	7.3 [6.4; 8.4]	8.7 [7.5; 10.0]
Injury from interpersonal assault	7.6 [6.3; 9.3]	8.9 [7.3; 10.9]

Notes: The within-individual comparisons were adjusted for age, calendar month and all time-stable individual-level unmeasured confounders (e.g., genetic risks, early childhood environmental factors, and early-onset neurodevelopmental disorders). The psychiatric disorders were defined hierarchically.

eTable 12. Sensitivity test: Testing for left truncation bias by including cohorts who were 12 years old or younger when the self-harm data were made available (Finland: 1975-2000; Sweden: 1961-1993)

	**All cohorts (reference)**	**No left truncation bias**
	**aOR [95% CI]**	**aOR [95% CI]**
**Schizophrenia-spectrum disorders**		
Fall-related injury	2.1 [1.8; 2.4]	2.0 [1.7; 2.4]
Transport-related injury	2.7 [2.1; 3.5]	2.2 [1.6; 3.1]
Traumatic brain injury	2.4 [2.0; 2.9]	2.3 [1.8; 2.8]
Injury from interpersonal assault	2.0 [1.6; 2.5]	1.7 [1.2; 2.3]
**Bipolar disorder**		
Fall-related injury	2.0 [1.6; 2.4]	2.0 [1.5; 2.5]
Transport-related injury	2.9 [2.1; 4.1]	2.5 [1.6; 3.8]
Traumatic brain injury	2.9 [2.3; 3.7]	2.9 [2.1; 4.1]
Injury from interpersonal assault	2.7 [1.9; 3.8]	2.1 [1.3; 3.4]
**Depression**		
Fall-related injury	2.4 [2.1; 2.6]	2.4 [2.1; 2.8]
Transport-related injury	2.4 [1.9; 2.9]	2.2 [1.8; 2.8]
Traumatic brain injury	2.6 [2.3; 3.0]	2.3 [2.0; 2.8]
Injury from interpersonal assault	2.7 [2.3; 3.3]	2.6 [2.1; 3.2]
**No psychiatric disorders**		
Fall-related injury	6.9 [6.2; 7.6]	6.8 [6.0; 7.7]
Transport-related injury	6.3 [5.4; 7.4]	6.1 [5.1; 7.5]
Traumatic brain injury	7.3 [6.4; 8.4]	6.5 [5.4; 7.8]
Injury from interpersonal assault	7.6 [6.3; 9.3]	8.9 [6.9; 11.4]

Notes: The within-individual comparisons were adjusted for age, calendar month and all time-stable individual-level unmeasured confounders (e.g., genetic risks, early childhood environmental factors, and early-onset neurodevelopmental disorders). The psychiatric disorders were defined hierarchically.

eTable 13. Sensitivity test: Excluding self-harm events of undetermined intent

	Any self-harm (reference)	Excluding self-harm events of undetermined intent
	**aOR [95% CI]**	**aOR [95% CI]**
**Schizophrenia-spectrum disorders**		
Fall-related injury	2.1 [1.8; 2.4]	1.8 [1.6; 2.1]
Transport-related injury	2.7 [2.1; 3.5]	2.6 [2.0; 3.5]
Traumatic brain injury	2.4 [2.0; 2.9]	2.1 [1.7; 2.5]
Injury from interpersonal assault	2.0 [1.6; 2.5]	1.5 [1.1; 2.1]
**Bipolar disorder**		
Fall-related injury	2.0 [1.6; 2.4]	1.9 [1.5; 2.3]
Transport-related injury	2.9 [2.1; 4.1]	2.7 [1.9; 3.9]
Traumatic brain injury	2.9 [2.3; 3.7]	2.7 [2.1; 3.5]
Injury from interpersonal assault	2.7 [1.9; 3.8]	2.3 [1.6; 3.4]
**Depression**		
Fall-related injury	2.4 [2.1; 2.6]	2.2 [2.0; 2.5]
Transport-related injury	2.4 [1.9; 2.9]	2.1 [1.7; 2.6]
Traumatic brain injury	2.6 [2.3; 3.0]	2.3 [2.0; 2.7]
Injury from interpersonal assault	2.7 [2.3; 3.3]	2.1 [1.7; 2.6]
**No psychiatric disorders**		
Fall-related injury	6.9 [6.2; 7.6]	3.0 [2.4; 3.8]
Transport-related injury	6.3 [5.4; 7.4]	2.8 [2.0; 4.0]
Traumatic brain injury	7.3 [6.4; 8.4]	3.7 [2.9; 4.8]
Injury from interpersonal assault	7.6 [6.3; 9.3]	3.9 [2.8; 5.6]

Notes: The within-individual comparisons were adjusted for age, calendar month and all time-stable individual-level unmeasured confounders (e.g., genetic risks, early childhood environmental factors, and early-onset neurodevelopmental disorders). The psychiatric disorders were defined hierarchically.
